# Sequential Dengue Virus Infection in Marmosets: Histopathological and Immune Responses in the Liver

**DOI:** 10.3390/v17121619

**Published:** 2025-12-15

**Authors:** Daniele Freitas Henriques, Livia M. N. Casseb, Milene S. Ferreira, Larissa S. Freitas, Hellen T. Fuzii, Carla Pagliari, Luciane Kanashiro, Paulo H. G. Castro, Gilmara A. Siva, Orlando Pereira Amador Neto, Valter M. Campos, Beatriz C. Belvis, Flavia B. dos Santos, Lilian R. M. de Sá, Pedro Fernando da Costa Vasconcelos

**Affiliations:** 1 Department of Arboviruses and Hemorrhagic Fevers, Evandro Chagas Institute, Ananindeua 67030-000, Brazil; 2 Postgraduate Program in Biology of Infectious and Parasitic Agents, Federal University of Pará, Belém 66075-110, Brazil; 3 Center of Tropical Medicine, Federal University of Pará, Belém 66055-240, Brazilhellenfuzii@gmail.com (H.T.F.); 4 State University of Pará, Belém 66050-540, Brazil; 5 Faculty of Medicine, University of Sao Paulo, Sao Paulo 01246-903, Brazil; cpagliari@usp.br (C.P.); luciane_kanashiro@yahoo.com.br (L.K.); 6 National Center of Primates, Ananindeua 67030-000, Brazil; 7 Department of Pathology, Evandro Chagas Institute, Ananindeua 67030-000, Brazil; 8 Hospital Santa Virgínia, São Paulo 03014-000, Brazil; 9 Viral Immunology Laboratory, Oswaldo Cruz Institute, Rio de Janeiro 21040-360, Brazil; 10 Faculty of Veterinary Medicine and zootechnics, University of São Paulo, São Paulo 05508-270, Brazil

**Keywords:** dengue virus (DENV), sequential infection, callithrix penicillata, liver pathology, immunohistochemistry

## Abstract

This study evaluated hepatic pathological and phenotypic alterations, along with the inflammatory response, following sequential dengue virus (DENV) infection in Callithrix penicillata, a relevant model for human endemic scenarios. Twenty-six animals were initially infected subcutaneously with DENV-3. Thirteen were euthanized between 1 and 7 days post-infection (dpi) to assess the acute phase, and up to 60 dpi for the convalescent phase. The remaining animals received a secondary DENV-2 infection two months later. Liver samples underwent histopathological and immunohistochemical analysis. Viral antigens were identified in hepatocytes, Kupffer cells, and Councilman bodies. Observed liver changes included apoptosis, lytic necrosis, midzonal inflammation, Kupffer cell hyperplasia and hypertrophy, sinusoidal dilation, and hemosiderin deposition. Both primary and secondary infections increased activated macrophages, NK cells, S-100 protein, and B lymphocytes. Primary infection was associated with elevated CD4^+^ T cells, IFN-γ, TGF-β, IL-10, and Fas expression, whereas secondary infection induced higher IFN-γ, TNF-α, IL-8, Fas, and VCAM levels. These findings mirror hepatic alterations in severe human dengue cases and underscore the role of direct viral effects and immune dysregulation in liver injury. The results support C. penicillata as a suitable non-human primate model for studying DENV pathogenesis.

## 1. Introduction

Over the past five decades, the global incidence of dengue has increased approximately 30-fold [1,2,3], particularly in tropical and subtropical regions where the virus is now hyperendemic due to the co-circulation of all four dengue virus (DENV) serotypes [4,5,6]. These four antigenically distinct but closely related serotypes (DENV-1 to DENV-4) [7] can cause a spectrum of clinical outcomes in humans, ranging from asymptomatic infection to severe and potentially fatal disease [8], with DENV-2 and DENV-3 more frequently associated with severe clinical manifestations [9].

During DENV infection in both humans and experimental models, viral tropism has been documented in multiple organs, including the spleen, liver, lymph nodes, kidneys, heart, lungs, thymus, bone marrow, and brain [10,11,12,13,14,15,16,17]. Histopathological alterations in these organs are often attributed to a dysregulated host immune response, driven by activation of cytotoxic T lymphocytes and excessive production of proinflammatory cytokines [16,18,19,20,21,22,23,24]. Elevated levels of IFN-γ, TNF-α, IL-8, IL-10, IL-18 (MIF), CCL2 (MCP-1), CCL4, CCL5 (RANTES), and CXCL10 (IP-10) have been associated with severe disease and are considered potential biomarkers for disease progression and severity [25,26,27,28,29,30,31,32,33,34,35,36].

One of the main challenges in studying dengue pathogenesis lies in the lack of experimental animal models that consistently reproduce human-like disease following inoculation with clinical viral isolates [37,38,39,40,41]. Investigations in wild reservoirs are also critical for understanding the long-term maintenance of viral transmission and the risk of human infection with newly emerging, genetically distinct DENV strains [42,43].

Animal models for DENV infection have important limitations, as many species develop only transient and low-level viremia, failing to reproduce human infection [44]. In contrast, studies in marmosets (Callithrix jacchus) have demonstrated consistent and high-titer viremia following inoculation with clinical human isolates (10^7^–10^3^ PFU), along with clinical signs such as fever, reduced activity, and hematological and biochemical alterations [45,46].

The detection of DENV in a Callithrix jacchus specimen that died with hemorrhagic manifestations during an epizootic in northeastern Brazil [47] highlights the importance of investigating sylvatic NHPs, especially given the potential emergence of enzootic cycles via human-to-wildlife spillback. In this context, Callithrix penicillata, a closely related species, represents an appropriate model for studying dengue pathogenesis in humans, as it has been shown to develop high viremia following DENV-3 infection, along with other clinical and laboratory findings consistent with dengue disease [48].

In this study, we aimed to characterize hepatic involvement and the associated immune response in Callithrix penicillata following sequential infection with DENV-3 and DENV-2, in order to model aspects of dengue pathogenesis observed in endemic human populations.

## 2. Materials and Methods

### 2.1. Ethical Statement

All procedures involving animals were approved by the Ethics Committee on Animal Research of the Evandro Chagas Institute (ECI) and the National Primate Center (CENP) under protocol number 0061/2009 (16 September 2009). Authorization for animal use and viral manipulation was granted by the Brazilian Institute of Environment and Renewable Natural Resources (IBAMA; protocol 22047—16 June 2010). Viral stocks used in this study are part of the ECI collection (protocol 006031/2013-91).

### 2.2. Virus Strains

DENV-2 (strain RNH H 744248) and DENV-3 (strain RNH H 712149) were originally isolated from the lymph node and liver, respectively, of fatal human dengue cases classified by the Brazilian Ministry of Health as dengue hemorrhagic fever/dengue shock syndrome (DHF/DSS). Viral identity and serotype were confirmed via reverse transcription polymerase chain reaction (RT-PCR), following the method described by Johnson et al. (2005) [49]. Viral stocks were propagated once in C6/36 mosquito cells [50], and serotype confirmation was performed by indirect immunofluorescence assay using serotype-specific monoclonal antibodies (Bio-Manguinhos/Fiocruz, Ministry of Health, BRA/Rio de Janeiro) [51].

### 2.3. Experimental Infection Protocol

A total of 26 Callithrix penicillata (black-tufted marmosets), aged between 1 and 10 years, were obtained from the breeding colony at CENP (Ananindeua, Pará, Brazil). All animals tested negative for anti-flavivirus antibodies prior to inclusion in the study. The animals were individually housed in stainless steel cages (0.80× 0.80× 0.90 m) with retractable floors, and were fed a standardized diet consisting of Megazoo P25 primate chow, supplemented with fruits, vegetables, tubers, milk, and eggs, along with ad libitum access to water, in accordance with CENP’s nutritional protocols.

Twenty-four animals were subcutaneously inoculated with 0.5 mL of culture medium containing DENV-3 (3.23 × 10^3^ PFU/mL) as primary infection (PI). Of these, 12 fasted animals (≥12 h fasting) were euthanized at defined time points: daily from 1 to 7 days post-infection (dpi) for the acute phase, and at 15, 20, 30, 45, and 60 dpi for the convalescent phase. Euthanasia was performed by intravenous administration of ketamine (15 mg/kg) and xylazine (1 mg/kg), followed by liver sample collection.

Two months after the primary infection (PI), the remaining 12 animals were subjected to secondary infection (SI) with 0.5 mL of DENV-2 (4.47 × 10^4^ PFU/mL), following the same inoculation and sampling protocol. Two additional non-infected animals were used as negative controls (NC) to provide baseline hepatic tissue for comparison; these animals were euthanized concurrently with the final sampling point of both the PI and SI at 60 days p.i.

### 2.4. Confirmation of DENV Infection in NHPs

Viral RNA detection and serological analyses in these non-human primates (NHPs) have been previously performed and described in a prior study [52]. Viral infection was confirmed by viral RNA detection via RT-qPCR and IgM seroconversion using the MAC-ELISA method. Total anti-DENV antibodies were also evaluated by hemagglutination inhibition assay [53]. The aforementioned experiment used the same group of callitrichids, inoculation doses, sampling points, and collection procedures that we analyzed in our study.

### 2.5. Histopathological Processing and Microscopic Evaluation

Liver samples were fixed in 10% buffered formalin for 24 h, then transferred to 70% ethanol for storage. Standard histological processing was performed through graded ethanol dehydration, culminating in absolute ethanol, followed by two immersions in xylene at room temperature. Samples were then embedded in paraffin through two 60 °C paraffin baths. Paraffin blocks were cooled and sectioned using a rotary microtome (Jung Histocut 820, Leica, Germany/Nussloch) to obtain 5 μm-thick sections for histopathological and immunohistochemical analysis.

The analyses were performed by two observers who were unaware of the identification of each case, using optical microscopy with magnifications of 40×, 100×, 200×, and 400×. A NiU model photomicroscope (Nikon, JP/Tóquio) with an attached DS-U3 digital camera (Nikon, JP/Tóquio) was used for photographic documentation.

### 2.6. Immunohistochemistry

Paraffin-embedded liver tissues were sectioned at 5 μm, dewaxed, and mounted onto high-adhesion microscope slides. Immunohistochemistry (IHC) was performed using the alkaline phosphatase method for the detection of DENV-specific antigens, employing polyclonal anti-DENV-2 and anti-DENV-3 antibodies produced at the Evandro Chagas Institute [53,54]. Negative controls were included in all assays.

Semi-quantitative analysis was performed using a light microscope (AXIO IMAGER Z1-ZEISS, model 4560006, Carl Zeiss, Oberkochen, Germany) equipped with a 1 cm^2^ graduated grid (area = 0.0625 mm^2^) under 400× magnification. Viral antigen-positive cells were counted in ten microscopic fields and scored using a 5-point scale: 0 = no positive cells; 1 = 1–2 positive cells; 2 = 3–5 positive cells; 3 = 6–10 positive cells; and 4 = >10 positive cells [55].

### 2.7. Histopathology

Liver tissue sections (5 μm) were dewaxed in xylene, rehydrated through graded ethanol, and stained with hematoxylin and eosin (H&E). Slides were examined under a light microscope (AXIO IMAGER Z1-ZEISS, model 4560006, Carl Zeiss, Oberkochen, Germany).

Microscopic evaluation included hepatocellular, structural, and circulatory alterations. Morphological diagnoses were made individually for each animal and categorized by infection phase (acute or convalescent) and infection type (PI or SI). A semi-quantitative scoring system was applied to assess lesion severity and distribution, using a 4-point scale: 0 = normal histology/absence of lesion; 1 = mild; 2 = moderate; and 3 = severe. Lesions were also mapped by liver zone: zone 1 (periportal), zone 2 (midzonal), and zone 3 (centrilobular).

To estimate overall hepatic injury per animal, the mean score across all evaluated parameters was calculated and compared to that of control animals. Scoring parameters and definitions are detailed in Table S1.

To assess whether the lesions observed in the groups could be characterized as hepatitis, the mean of the summed scores for three main histological parameters was calculated: cell death (hepatocyte apoptosis and necrosis, score = 3), inflammation (acinar, portal tract, and central vein, score = 2), and cellular swelling (score = 1). Each parameter was assigned a specific score, adapted from the methodology of Ishak et al. (1995) [56], taking into account the pathological significance of each event in the progression of hepatitis [57,58,59,60].

### 2.8. Immunophenotyping and Cytokine Expression in Liver Tissue

Hepatic immune cell phenotyping and cytokine expression were assessed via IHC following established protocols [17]. Briefly: (I) Sections were dewaxed in xylene and rehydrated through decreasing concentrations of ethanol; (II) Endogenous peroxidase activity was blocked using 3% hydrogen peroxide in the dark; (III) Antigen retrieval was performed at 95 °C using citrate buffer (pH = 6.0, 7.2, or 9.0), depending on antibody requirements; (IV) Non-specific protein binding was blocked using 10% skim milk in distilled water; (V) Sections were incubated overnight at 4 C with primary antibodies diluted in 1% bovine serum albumin (BSA); (VI) After washing, sections were incubated with a secondary antibody specific to the host species of the primary antibody; (VII) Detection was performed using either a streptavidin-biotin complex or a polymer-based system, as previously standardized; (VIII) Visualization was achieved with 3,3-diaminobenzidine (DAB) prepared in PBS and hydrogen peroxide; (IX) Counterstaining was done with hematoxylin; (X) Dehydration was completed through graded ethanol, and slides were mounted using Permount™ resin. Washing steps between procedures used running and/or distilled water. Positive controls included human lymph node and Callithrix jacchus tissues. Primary antibody dilutions are provided in Table S2.

Immunohistochemical reactions were quantified by manually counting immunolabeled cells using a binary criterion of positivity. A cell was classified as positive when it displayed a clearly discernible brown DAB precipitate in the cytoplasm or nucleus, consistent with the expected staining pattern for each antibody. Weak, diffuse, or pattern-incompatible signals were excluded. Human lymph node tissue and Callithrix jacchus tissue served as positive controls, accompanied by samples from two non-infected animals as negative controls.

Cell counts were performed using a light microscope equipped with a graded reticle (0.0625 mm^2^ area) at 400× magnification. For each marker, 10 fields were quantified in zone 1 (periportal), 8–10 fields in zone 2 (midzonal), and 10 fields in zone 3 (centrilobular), in addition to 5 portal tracts and 5 central veins. For each animal, the mean number of positive cells per field was calculated and subsequently converted to cells/mm^2^. Individual means were then grouped according to infection phases (Ag.1, Conv.1, Ag.2, and Conv.2).

### 2.9. Statistical Analysis

Histopathological data were analyzed using one-way analysis of variance (ANOVA) followed by Tukey’s post hoc test. For immune response quantification, two-way ANOVA was applied to account for the two factors “Infection Phase” (Acute vs. Convalescent) and “Infection Type” (Primary vs. Secondary), followed by Bonferroni post-test correction was applied. Statistical significance was set at *p* ≤ 0.05. Correlation analyses (*p* < 0.05) were used to identify potential interactions between immunological markers [61]. All analyses and graph generation were performed using GraphPad Prism version 6.0 for Windows (GraphPad Software, San Diego, CA, USA).

## 3. Results

### 3.1. Detection of Viral Antigen in Liver Tissue

**Figure 1:**
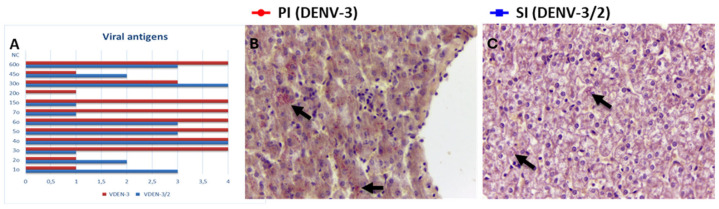
Detection and expression of viral antigen in the liver of PNH. (**A**) Detection of viral antigens in the liver of Callithrix penicillata experimentally infected by DENV-3 and DENV-3/2. (**B**) Immunostaining in the liver tissue of marmosets infected with DENV-3 at 60 dpi (400×). (**C**) Immunostaining in liver tissue from marmosets infected with DENV-3/2 60 dpi (400×). **Legend:** 0 = absence of antigens; 1 = presence of one to two cells expressing viral antigens; 2 = presence of 3–5 cells expressing viral antigens; 3 = presence of 6–10 cells expressing specific antigens; and 4 = presence of more than 10 cells expressing specific antigens in an area occupied by 10 histological fields of 400×.

Viral antigens were detected in liver tissues of all DENV-infected marmosets during both PI (DENV-3) and SI (DENV-2), with the exception of one animal at 20 dpi after SI (Figure 1A). Although this animal did not exhibit detectable antigen in liver tissue, serological testing confirmed a monotypic IgM response to DENV-2 and a cross-reactive total antibody response, as previously reported [48].

Immunohistochemistry revealed variable levels of viral antigen expression, ranging from mild (1–2 antigen-positive cells per field) to intense (>10 antigen-positive cells per field). The most pronounced antigen expression was observed during the acute phase of PI, with localization predominantly in hepatic zones 2 and 3 (midzonal and centrilobular regions, respectively) (Figure 1A–C).

### 3.2. Liver Histopathological Characterization Following Primary and Sequential Dengue Virus Infections

**Figure 3:**
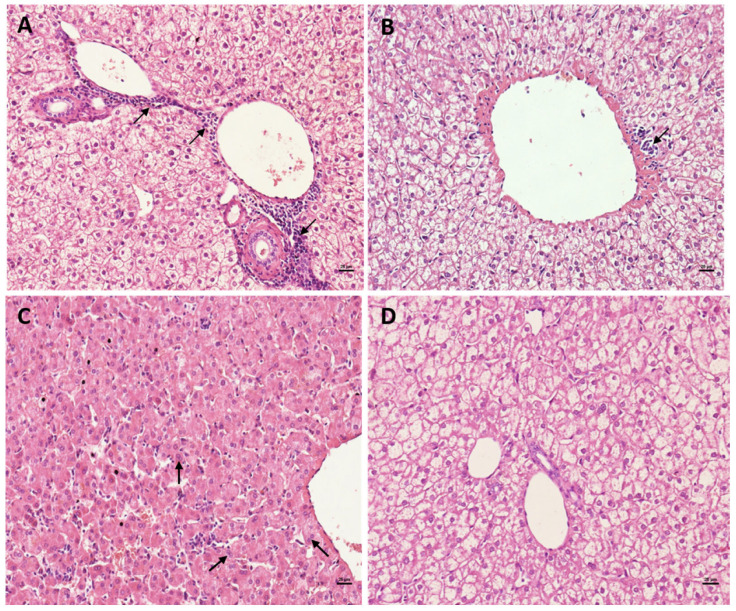
Liver of Callithrix penicillata infected with DENV. (**A**) Liver showing inflammatory infiltrate in the portal space (arrow), primary 5 dpi, HE, 400×. (**B**) Liver showing inflammatory infiltrate in the central hepatic vein (arrow), primary 5 dpi, HE, 200×. (**C**) Liver showing regenerative hepatocyte alteration, binucleate regeneration (arrow), primary 60 dpi, HE, 200×. (**D**) Histology of the liver of sentinel animal 2, HE, 200×.

Histopathological analysis of hepatic tissues from non-human primates revealed distinct patterns of liver injury following primary infection (PI) with DENV-3 and sequential secondary infection (SI) with DENV-2. Two non-infected primates, used as negative controls and euthanized at kinetic time points corresponding to the PI and SI, showed only rare foci of inflammatory cells (≤3 foci, composed of lymphocytes and macrophages) and mild to moderate diffuse hepatocellular swelling (Figure 3D), with no evidence of necrosis, apoptosis, steatosis, or marked alterations of Kupffer cells. These findings established the baseline pattern for the species and allowed the lesions observed in infected animals to be attributed to viral and immune responses.

**Figure 2:**
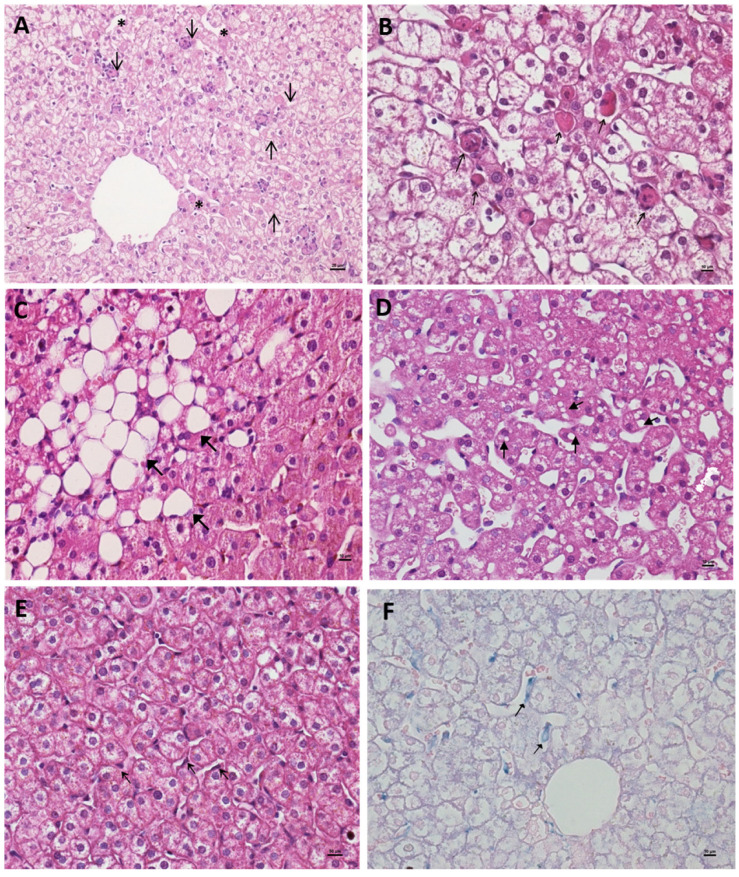
Liver of Callithrix penicillata infected with DENV. (**A**) Liver showing cell death (Councilman bodies and lytic necrosis) of hepatocytes (arrows) and sinusoid dilatation (*), 2 dpi primary, HE, 200×. (**B**) Liver showing cell death (Councilman bodies and lytic necrosis) of hepatocytes (arrows), 6 dpi primary, HE, 400×. (**C**) Liver showing macrovesicular steatosis in hepatocytes (arrows), 3 dpi primary, HE, 400×. (**D**) Liver showing microvesicular steatosis in hepatocytes (arrows), 15 dpi secondary, HE, 400×. (**E**) Liver showing hyperplasia/hypertrophy of Kupffer cells (arrows), 2 dpi secondary, HE, 400×. (**F**) Liver showing hemosiderin in Kupffer (arrows), 5 dpi primary, Perls, 400×.

During PI, lesions were characterized by multifocal hepatocellular damage, generally of mild to moderate intensity, with a predilection for zones 2 and 3 of the hepatic acinus. Lytic necrosis was a prominent feature observed from 1 to 6 days post-infection (dpi) (Figure 2A,B), and again at 15 and 60 dpi. Apoptotic bodies (Councilman bodies) were also frequently identified in the early acute phase (1–6 dpi) (Figure 2A,B). Macrovesicular steatosis was present at 3 and 60 dpi (Figure 2C), and hepatocellular swelling was consistently observed throughout the entire study period (1–60 dpi). Kupffer cells exhibited signs of hyperplasia and hypertrophy between 1 and 5, 20, and 60 dpi, along with hemosiderin accumulation particularly from 3 to 6 dpi (Figure 2F). Inflammatory infiltrates composed of lymphocytes, neutrophils, macrophages, and megakaryocytes were evident in the hepatic parenchyma from 1 to 15 dpi and at 60 dpi (Figure 3B), as well as in the portal tracts from 1 to 7 dpi and again at 45 dpi (Figure 3A). Signs of tissue regeneration began to appear around 20 dpi (Figure 3C), indicating a shift toward hepatic recovery.

Following SI with DENV-2, many histopathological features overlapped with those observed in PI, including lytic necrosis (noted from 1 to 7 dpi, and again at 30 and 60 dpi), steatosis (2 and 15 dpi) (Figure 2D), hepatocyte swelling (persisting across all time points), and Kupffer cell alterations (hyperplasia, hypertrophy, and hemosiderin deposition observed from 2 to 7 dpi and at 45 dpi) (Figure 2E). Inflammatory responses were again prominent, with infiltrates found in both acinar and portal regions from early acute through late stages (1–60 dpi). However, a notable difference in SI was the absence of apoptotic bodies, suggesting a distinct pattern of cellular injury compared to PI. Furthermore, lesions during SI were predominantly localized to zone 2 and persisted until the end of the experimental timeline.

Quantitative comparisons between the two infection phases revealed that certain histopathological changes were significantly more pronounced in PI. Apoptosis (*p* < 0.007), centrilobular inflammation (*p* < 0.050), hemosiderin deposition (*p* < 0.027), and Kupffer cell hyperplasia/hypertrophy (*p* < 0.047) were all significantly more prevalent following PI than SI, suggesting a more cytopathic or immunologically aggressive profile in response to primary DENV-3 infection. These findings highlight both shared and distinct pathological mechanisms involved in liver injury during sequential dengue virus infections.

### 3.3. Hepatitis Characterization in Primary (DENV-3) and Secondary (DENV-3/2) Infections

**Figure 4:**
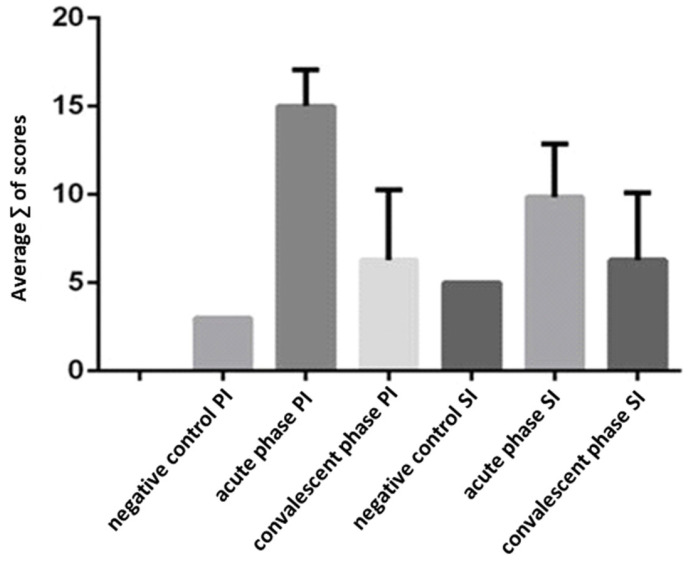
Characterization of hepatitis in the primary and secondary infection phases. Legend: 0 to 20 = mean of the sum of the scores of some parameters evaluated during histopathological analysis (apoptosis, necrosis, acinar inflammation, PT inflammation and cellular swelling). Negative control PI = control animal of primary infection; Acute phase PI = acute phase of primary infection; Convalescent phase PI = convalescent phase of primary infection; Negative control SI= control animal of secondary infection; Acute phase SI = acute phase of secondary infection; Convalescent phase SI = convalescent phase of secondary infection.

A comparative analysis of key histopathological parameters associated with hepatitis revealed statistically significant differences between animals in the acute phase of both primary (PI) and secondary (SI) infections when compared to uninfected controls (*p* < 0.000). In contrast, no significant alterations were observed in animals during the convalescent phase of either infection (Figure 4). These findings support the characterization of acute hepatitis in non-human primates during the early stages of both DENV-3 (PI) and sequential DENV-2 (SI) infections. However, hepatic tissue from convalescent animals did not show histopathological profiles consistent with active hepatitis. Notably, the mean composite score was highest in animals during the acute phase of PI, reflecting numerically more pronounced hepatic changes in response to primary DENV-3 infection, although this difference was not statistically significant compared to acute SI (Figure 4).

### 3.4. Characterization of Hepatic Immunophenotype and Cytokine Profile in Non-Human Primates Following Sequential Dengue Virus Infection

**Figure 5:**
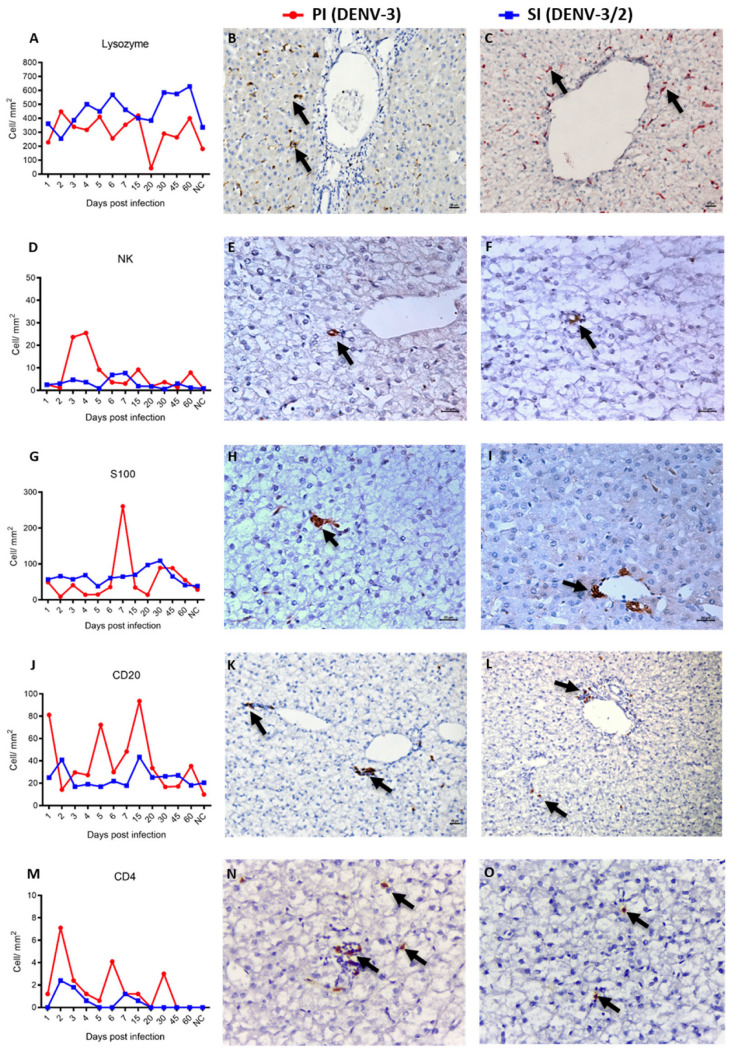
Immunohistochemical of liver parenchyma of NHP of the species Callithrix penicillata infected with DENV. (**A**) Quantification of lysozyme immunoexpression during PI and SI kinetics. Immunostaining for Lysozyme (arrow), (**B**) 4 dpi primary, IHC, 200× and (**C**) 4 dpi secondary, IHC, 200×; (**D**) Quantification of NK cell immunoexpression during PI and SI kinetics. Immunostaining for NK cells (arrow), (**E**) 5 dpi primary, IHC, 400× and (**F**) 4 dpi secondary, IHC, 400×; (**G**) Quantification of antigen-presenting cell immunoexpression, S100+ protein during PI and SI kinetics. Immunostaining for antigen-presenting cells, S100+ protein (arrow), (**H**) 3 dpi primary, IHC, 400× and (**I**) 4 dpi secondary, IHC, 400×; (**J**) Quantification of the immunoexpression of CD20^+^ B lymphocytes during the kinetics of PI and SI. Immunostaining for CD20^+^ B lymphocytes (arrow), (**K**) 7 dpi primary, IHC, 200× and (**L**) 4 dpi secondary, IHC, 200×; (**M**) Quantification of the immunoexpression of CD4^+^ T lymphocytes during the kinetics of PI and SI. Immunostaining for CD4^+^ T lymphocytes (arrow), (**N**) 4 dpi primary, IHC, 400× and (**O**) 4 dpi secondary, IHC, 400×. All data points represent individual animals, with no averaging. Each symbol denotes one animal.

Histopathological lesions in the liver of non-human primates subjected to sequential DENV infection were accompanied by expression of apoptosis markers, inflammatory cell infiltrates, and cytokines (Table S3). Lysozyme, a marker of activated macrophages, was detected in all infected animals and showed significantly higher expression during the convalescent phase of secondary infection (SI) (*p* < 0.01) (Figure 5A–C). Conversely, NK cell infiltration was more prominent during the acute phase of primary infection (PI) (*p* < 0.01) (Figure 5D,E), with significantly higher frequencies in the acute phase of PI (Ac1) compared to both the convalescent phase (Conv1) (*p* < 0.01) and the acute phase of SI (Ac2) (*p* < 0.01) (Figure 5F).

No significant differences were observed in S-100 protein expression when comparing PI and SI overall. However, elevated expression was detected in the acute phase of PI and in the convalescent phase of SI (*p* < 0.01) (Figure 5G–I). CD20^+^ and CD4^+^ lymphocytes were also more frequently detected in the liver during PI compared to SI (*p* < 0.01) (Figure 5J–M). CD4^+^ cells were absent in negative controls, suggesting virus-induced recruitment. CD20^+^ cells were observed across both acute and convalescent phases of PI and SI, albeit in significantly lower numbers in control animals (*p* < 0.01) (Figure 5K,L).

**Figure 6:**
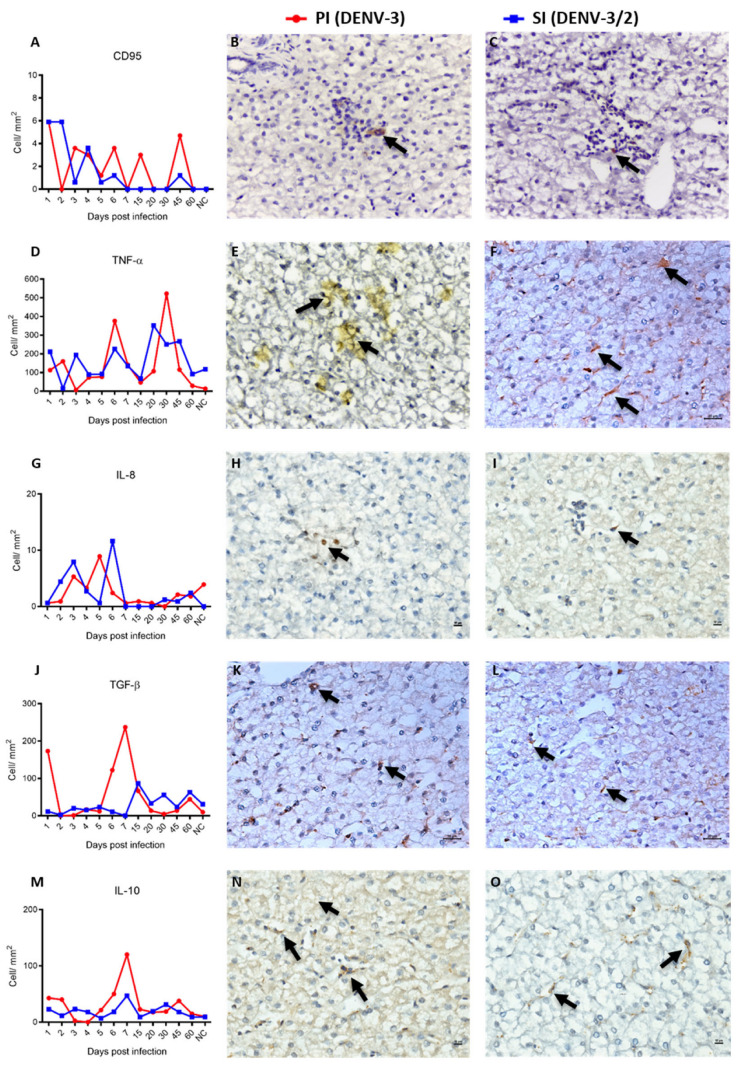
Immunohistochemical of liver parenchyma of NHP of the species *Callithrix penicillata* infected with DENV. (**A**) Quantification of Fas-CD95+ protein immunoexpression during PI and SI kinetics. Immunostaining for Fas-CD95+ protein (arrow), (**B**) 1 dpi primary, IHC, 400× and (**C**) 4 dpi secondary, IHC, 400×; (**D**) Quantification of TNF-α immunoexpression during PI and SI kinetics. Immunostaining for TNF-α (arrow). (**E**) 7 dpi primary, IHC, 400× and (**F**) 4 dpi secondary, IHC, 400×; (**G**) Quantification of IL-8 immunoexpression during PI and SI kinetics. Immunostaining for IL-8 (arrow), (**H**) 45 dpi primary, IHC, 400× and (**I**) 4 dpi secondary, IHC, 400×; (**J**) Quantification of TGF-β immunoexpression during PI and SI kinetics. Immunostaining for TGF-β (arrow), (**K**) 7 dpi primary, IHC, 400× and (**L**) 15 dpi secondary, IHC, 400×; (**M**) Quantification of IL-10 immunoexpression during PI and SI kinetics. Immunostaining for IL-10 (arrow), (**N**) 1 dpi primary, IHC, 400× and (**O**) 2 dpi secondary, IHC, 400×. All data points represent individual animals, with no averaging. Each symbol denotes one animal.

CD95^+^ (Fas receptor) cell frequencies were comparable between PI and SI (Figure 6A–C). Nonetheless, Fas protein expression was significantly elevated during the acute phases of both PI and SI relative to controls (*p* < 0.01). These cells were predominantly distributed in zone 2 (Z2) during the acute phase of PI (Figure 6B).

Regarding cytokine expression, TNF-α levels did not differ significantly between PI and SI. However, during SI, TNF-α expression was significantly higher in the convalescent phase compared to the acute phase (*p* < 0.01), a pattern not statistically significant in PI (Figure 6D–F). Similarly, IL-8 showed a consistent expression profile across infections, with significant differences between acute and convalescent phases in both PI and SI (*p* < 0.01) (Figure 6G–I). TGF-β and IL-10 were markedly upregulated during the acute phase of PI when compared to SI and controls (*p* < 0.01), suggesting a robust anti-inflammatory response induced by DENV-3 (Figure 6J–O).

**Figure 7:**
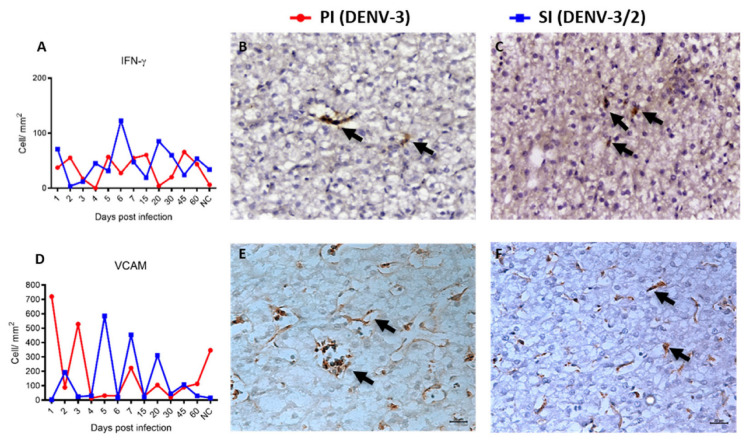
Immunohistochemical of liver parenchyma of NHP of the species Callithrix penicillata infected with DENV. (**A**) Quantification of IFN-γ immunoexpression during PI and SI kinetics. IFN-γ immunostaining (arrow), (**B**) 15 dpi primary, IHC, 400× and (**C**) 4 dpi secondary, IHC, 400×; (**D**) Quantification of VCAM immunoexpression during PI and SI kinetics. VCAM immunostaining (arrow), (**E**) 3 dpi primary, IHC, 400× and (**F**) 4 dpi secondary, IHC, 400×. All data points represent individual animals, with no averaging. Each symbol denotes one animal.

No significant differences in IFN-γ or VCAM expression were detected between PI and SI (Figure 7A–F). However, IFN-γ levels were significantly higher in both phases of infection when compared to their respective controls (*p* < 0.01). VCAM expression was elevated in both acute and convalescent phases of SI relative to controls (*p* < 0.01), but not during PI. An illustration of a negative control is included in Figure S1.

## 4. Discussion

Histopathological lesions in the liver associated with DENV infection are usually reported in severe dengue cases. To date, there is limited information regarding liver histopathology during classic dengue or in New World nonhuman primates (NHPs). Moreover, the detection of DENV in sylvatic NHPs [47,62] and the potential emergence of a sylvatic dengue cycle in the Americas through spillback from humans underscore the importance of investigating infection patterns in these animals.

In this study, we employed epidemic, non-adapted DENV strains originally isolated from lymph node and liver tissues of a fatal severe dengue case in Brazil, demonstrating that these clinical isolates retain the ability to replicate in vivo in NHPs and induce infection. Furthermore, histopathological changes in the livers of Callithrix penicillata infected with DENV resembled those reported in human fatal dengue cases [10,63,64,65,66,67,68,69,70], albeit with differing intensity and scope.

During the first seven days post-primary infection (PI), hepatocyte apoptosis was the predominant form of cell death observed, consistent with findings from a study on livers of 17 fatal dengue hemorrhagic fever cases in Brazil [69]. Viral antigen was detected in Councilman bodies, indicating a direct viral contribution to liver lesions. Concurrently, increased expression of apoptosis-associated cytokines TGF-β and TNF-α was observed during the acute phase of PI [71,72,73,74,75], highlighting their potential role in hepatocyte apoptosis.

Councilman bodies were not observed in sequential infections (SI), likely due to rapid clearance kinetics preventing their detection via histopathology. Pre-existing antibodies from the primary infection may have accelerated immune-mediated clearance of infected hepatocytes before Councilman bodies became morphologically apparent, although this remains speculative. Fas (CD95^+^) protein expression was significantly elevated during the acute phase of SI compared to controls. Discrete foci of lytic necrosis, predominantly distributed in zone 2 (Z2), were present across all infection phases in both PI and SI. Although the cause of lytic necrosis in dengue is multifactorial, the absence of clinical or laboratory markers typical of severe dengue (e.g., plasma leakage) in the NHPs suggests that the observed necrosis may be primarily due to virus-cell interactions impairing hepatocyte function [52,76,77]. Moreover, the consistent association of necrotic areas with lymphocytic infiltration, macrophages, and neutrophils suggests a potential contribution of cytotoxic T lymphocytes and other immune cells to hepatocyte injury, although functional experiments would be required to confirm their specific roles.

Hepatic steatosis, a common finding in severe dengue cases [63,64,67,69], was also observed in this study, though less frequently, with predominance in Z2. The development of steatosis has been linked to host factors and viral infection [78,79]. Notably, NHPs exhibiting steatosis during PI (3 and 60 dpi) and SI (2 and 15 dpi) showed moderate to intense DENV antigen presence in Z2, suggesting that viral infection may disrupt lipid homeostasis [80].

Cellular swelling, a typical liver injury during human DENV infections [65,67,77], was prevalent during both acute and convalescent phases of PI and SI, mirroring observations in DENV-2-infected adult BALB/c mice [81]. Kupffer cell hyperplasia/hypertrophy and lysozyme expression were significantly increased in infected NHPs compared to controls during both PI and SI, suggesting a potential response to viral infection and immune activation, although functional experiments would be required to confirm the precise role of these macrophages. Despite prominent liver injury during the acute phases of PI and SI, no significant differences were detected between these infections.

Our findings reveal comparable liver damage in Callithrix penicillata following sequential DENV-3 and DENV-2 infections. The similar injury intensity during both infections suggests that heterotypic secondary infection neither exacerbated liver pathology nor conferred effective protection to prevent histological alterations during SI. These hepatic changes in marmosets may parallel those in human dengue fever, where mild to moderate elevations of AST and ALT are common [82,83,84,85]. In NHPs, transaminase increases were observed during PI, with AST rising during SI [52], consistent with human dengue patterns showing higher AST relative to ALT levels [86,87].

More pronounced liver damage in the acute phase appears to coincide with higher antigen detection compared to the convalescence. The relationship between liver injury severity, DENV antigen frequency, and cellular and cytokine immune responses suggests a potential contribution of both viral cytopathic effects and localized inflammatory responses, potential contribution of both.

Lytic necrosis and inflammatory infiltrates in infected NHPs predominantly localized to Z2, although other lesions did not display strict zonal preference, despite being most frequently observed in Z2 followed by zone 3 (Z3). Similar zonal distributions have been reported in severe human dengue cases [15,20,63,64,65,69,88]. While differential blood flow and hepatocyte heterogeneity across acinar zones are established [89], the factors governing DENV-associated lesion tropism to Z2 remain speculative.

Although apoptosis was more prevalent than necrosis in acute PI, the mild parenchymal involvement was consistent with relatively low inflammatory infiltrate. The necrosis and inflammation localized in Z2 may reflect immune cell recruitment toward clearing damaged hepatocytes, and apoptosis-mediated neutrophil migration through Fas receptor activation and chemokine synthesis could influence the density of infiltrate [90,91]. Councilman bodies were observed adjacent to necrotic and inflammatory foci, whereas Fas expression during SI was noted near necrotic foci only when Councilman bodies were absent—a distribution pattern reported in human fatal dengue [15,64,69].

Although liver lesions correlated with inflammatory infiltrates, the observed TGF-β expression suggests it may have contributed to limiting inflammation due to its anti-inflammatory and pro-apoptotic roles. This pattern of expression was predominantly observed during the acute phase of PI, which is consistent with reports from fatal yellow fever and dengue cases [20,54,88,92,93].

Increased hepatic NK cells were detected in infected NHPs, paralleling findings in severe human dengue [15,88]. Experimental studies demonstrate that NK cells mediate cytolysis and induce hepatocyte apoptosis [94,95]. In our study, NK cell infiltration peaked between 3 and 4 dpi in PI, coinciding with peak IFN-γ expression at 2–5 dpi.

NK-derived IFN-γ may contribute to macrophages activation; accordingly, elevated IFN-γ, Kupffer cells, and TNF-α positivity were observed in infected NHPs. Kupffer cell activation and TNF-α expression appeared were more pronounced during SI, possibly reflecting heightened immune activation. In severe human dengue, Kupffer cell-associated TNF-α has been implicated in disease pathogenesis [16,88]. TNF-α is known to promotes recruitment and activation of neutrophils and monocytes to infection sites of infection [76]. Neutrophils were present throughout infection, with increased numbers in acute PI and convalescent SI. IL-8 prevalence during SI convalescence may suggest an immune cascade initiated by IFN-γ and Kupffer cell activity. TNF-α can also induces endothelial expression of adhesion molecules, potentially facilitating leukocyte recruitment [76]. VCAM-1 expression was elevated during SI but not PI, which may be influenced by TNF-α-mediated endothelial modulation, as previously reported [96,97,98,99,100].

A key limitation of this study is the lack of functional validation of the proposed immune mechanisms, which precludes direct attribution of causality to specific cell types or cytokines. The small sample size at each time point (*n* = 1) limits statistical power, although it allowed detailed kinetic analyses. As the study was conducted in Callithrix penicillata, the number of animals was necessarily restricted due to ethical and regulatory considerations associated with non-human primate experimentation. Standardizing of certain human antibodies, including anti-CD8 and IL-4, was also limited, constraining the evaluation of Th2 responses and the role of CD8+ T cells.

These limitations affect data interpretation, reducing statistical robustness and preventing causal attribution to specific immune pathways. Furthermore, such restrictions may limit the generalization of the findings beyond this primate model. Thus, the proposed mechanisms should be considered correlative, and future functional studies will be necessary to confirm them.

Nevertheless, sustained IFN-γ expression, together with increased Kupffer cells and IL-10 (most pronounced during the early phase of infection), suggests a predominantly Th1 immune profile in the liver, consistent with observations in human dengue patients [33,88,101].

Semi-quantitative morphological analysis revealed only acute hepatitis in infected animals, with significant differences between acute PI and SI versus controls. The presence of acute hepatitis during the early phases aligns with elevated ALT and AST levels previously reported in these NHPs [48]. The restriction of hepatitis to acute infection phases reinforces dengue’s acute and self-limiting nature. Examination of convalescent phases was crucial for detecting residual viral antigen, characterizing lesion resolution, inflammation, and regenerative hepatocyte changes such as binucleation observed exclusively post-PI, likely linked to the preceding acute injury.

## 5. Conclusions

Callithrix penicillata has been demonstrated to be a reliable experimental model for investigating sequential DENV infections. Infection with DENV-3 followed by DENV-2 in these primates results in mild acute hepatitis. The immunopathological features observed in the liver of Callithrix penicillata resemble those reported in human fatal dengue cases, suggesting that similar hepatic alterations may occur during mild dengue infections in humans. These findings generate hypotheses for future clinically relevant studies aimed at understanding the mechanisms underlying hepatic involvement and immune responses during dengue.

## Data Availability

Data can be requested from the corresponding author.

## Supplementary Materials

The following supporting information can be downloaded at: https://www.mdpi.com/article/10.3390/v17121619/s1. Table S1: Score parameters and characterization applied to the morphological analysis of the liver; Table S2: Antibodies used for the characterization of the immunophenotype and cytokine expression on the liver of Callithrix penicillata after sequential DENV infection; Table S3: Immunohistochemical evaluation—mean and standard deviation of the number of positive cells/mm2 in the hepatic acinus by group. Figure S1: Immunohistochemistry for the various markers in the hepatic parenchyma of the negative control.

## References

[1] Silva E.M.d., Oliveira G.V., Brito P.H.A.d., Consolin E.M., Brito P.F., Franco M.C., Melo L.V., Bordon L.B., Sena A.L., Lima V.O. (2024). A ocorrência de dengue no Brasil na última década: Avanços e desafios. Braz. J. Health Rev..

[2] Katzelnick L.C., Coloma J., Harris E. (2017). Dengue: Knowledge gaps, unmet needs, and research priorities. Lancet Infect. Dis..

[3] Yang X., Quam M.B.M., Zhang T., Sang S. (2021). Global burden for dengue and the evolving pattern in the past 30 years. J. Travel Med..

[4] Sharp T.M., Tomashek K.M., Read J.S., Margolis H.S., Waterman S.H. (2017). A New Look at an Old Disease: Recent Insights into the Global Epidemiology of Dengue. Curr. Epidemiol. Rep..

[5] Hamel R., Surasombatpattana P., Wichit S., Dauvé A., Donato C., Pompon J., Vijaykrishna D., Liegeois F., Vargas R.M., Luplertlop N. (2019). Phylogenetic analysis revealed the co-circulation of four dengue virus serotypes in Southern Thailand. PLoS ONE.

[6] Aring B.J., Gavali D.M.B., Kateshiya P.R., Gadhvi H.M., Mullan S., Trivedi K.K., Nathametha A.A., Nakhava A.H. (2021). Co-circulation of all the Four Serotype of Dengue Virus in Endemic Region of Saurashtra, Gujarat during 2019-2020 Season. J. Clin. Diagn. Res..

[7] Calisher C.H., Karabatsos N., Dalrymple J.M., Shope R.E., Porterfield J.S., Westaway E.G., Brandt W.E. (1989). Antigenic Relationships between Flaviviruses as Determined by Cross-neutralization Tests with Polyclonal Antisera. J. Gen. Virol..

[8] World Health Organization (2009). Dengue: Guidelines for Diagnosis, Treatment, Prevention and Control.

[9] Nisalak A., Endy T.P., Nimmannitya S., Kalayanarooj S., Thisayakorn U., Scott R.M., Burke D.S., Hoke C.H., Innis B.L., Vaughn D.W. (2003). Serotype-specific dengue virus circulation and dengue disease in bangkok, thailand from 1973 to 1999. Am. J. Trop. Med. Hyg..

[10] Burke T. (1968). Dengue haemorrhagic fever: A pathological study. Trans. R. Soc. Trop. Med. Hyg..

[11] Rosen L., Shroyer D.A., Tesh R.B., Freier J.E., Lien J.C. (1983). Transovarial Transmission of Dengue Viruses by Mosquitoes: Aedes albopictus and Aedes aegypti. Am. J. Trop. Med. Hyg..

[12] Jessie K., Fong M.Y., Devi S., Lam S.K., Wong K.T. (2004). Localization of Dengue Virus in Naturally Infected Human Tissues, by Immunohistochemistry and In Situ Hybridization. J. Infect. Dis..

[13] Paes M.V., Lenzi H.L., Nogueira A.C.M., Nuovo G.J., Pinhão T., Mota E.M., Basílio-De-Oliveira C.A., Schatzmayr H., Barth O.M., De Barcelos Alves A.M. (2009). Hepatic damage associated with dengue-2 virus replication in liver cells of BALB/c mice. Lab. Investig..

[14] Salgado D.M., Eltit J.M., Mansfield K.D., Panqueba C., Castro D., Vega M.R., Xhaja K.S., Schmidt D., Martin K.J., Allen P.D. (2010). Heart and Skeletal Muscle Are Targets of Dengue Virus Infection. Pediatr. Infect. Dis. J..

[15] Póvoa T.F., Alves A.M.B., Oliveira C.A.B., Nuovo G.J., Chagas V.L.A., Paes M.V. (2014). The Pathology of Severe Dengue in Multiple Organs of Human Fatal Cases: Histopathology, Ultrastructure and Virus Replication. PLoS ONE.

[16] Póvoa T.F., Oliveira E.R.A., Basílio-De-Oliveira C.A., Nuovo G.J., Chagas V.L.A., Salomão N.G., Mota E.M., Paes M.V. (2016). Peripheral Organs of Dengue Fatal Cases Present Strong Pro-Inflammatory Response with Participation of IFN-Gamma-, TNF-Alpha- and RANTES-Producing Cells. PLoS ONE.

[17] Hsu A.Y., Ho T., Lai M., Tan S.S., Chen T., Lee M., Chien Y., Chen Y., Perng G.C. (2019). Identification and characterization of permissive cells to dengue virus infection in human hematopoietic stem and progenitor cells. Transfusion.

[18] Costa V.V., Fagundes C.T., Souza D.G., Teixeira M.M. (2013). Inflammatory and innate immune responses in dengue infection: Protection versus disease induction. Am. J. Pathol..

[19] Guabiraba R., Ryffel B. (2014). Dengue virus infection: Current concepts in immune mechanisms and lessons from murine models. Immunology.

[20] Pagliari C., Quaresma J.A.S., Fernandes E.R., Stegun F.W., Brasil R.A., de Andrade H.F., Barros V., Vasconcelos P.F.C., Duarte M.I.S. (2013). Immunopathogenesis of dengue hemorrhagic fever: Contribution to the study of human liver lesions. J. Med. Virol..

[21] Ahsan J., Ahmad S.Q., Rafi T. (2018). Postmortem Findings In Fatal Dengue Haemorrhagic Fever. J. Coll. Physicians Surg. Pak..

[22] Khanam A., Gutiérrez-Barbosa H., Lyke K.E., Chua J.V. (2022). Immune-Mediated Pathogenesis in Dengue Virus Infection. Viruses.

[23] Yong Y.K., Wong W.F., Vignesh R., Chattopadhyay I., Velu V., Tan H.Y., Zhang Y., Larsson M., Shankar E.M. (2022). Dengue Infection—Recent Advances in Disease Pathogenesis in the Era of COVID-19. Front. Immunol..

[24] Tin E.N., Komarasamy T.V., Adnan N.A.A., Balasubramaniam V.R.M.T. (2025). Dengue virus infection: Immune response and thera-peutic targets. Am. J. Trop. Med. Hyg..

[25] Green S., Pichyangkul S., Vaughn D.W., Kalayanarooj S., Nimmannitya S., Nisalak A., Kurane I., Rothman A.L., Ennis F.A. (1999). Early CD69 Expression on Peripheral Blood Lymphocytes from Children with Dengue Hemorrhagic Fever. J. Infect. Dis..

[26] Weg C.A.M.V.D., Pannuti C.S., De Araújo E.S.A., Ham H.-J.V.D., Andeweg A.C., Boas L.S.V., Felix A.C., Carvalho K.I., De Matos A.M., Levi J.E. (2013). Microbial Translocation Is Associated with Extensive Immune Activation in Dengue Virus Infected Patients with Severe Disease. PLoS Neglected Trop. Dis..

[27] Solórzano V.E.F., Faria N.R.d.C., dos Santos C.F., Corrêa G., Cipitelli M.d.C., Ribeiro M.D., de Souza L.J., Damasco P.V., da Cunha R.V., dos Santos F.B. (2021). Different Profiles of Cytokines, Chemokines and Coagulation Mediators Associated with Severity in Brazilian Patients Infected with Dengue Virus. Viruses.

[28] Iyer S., Sucila T.G. (2022). Pathophysiologic and Prognostic Role of Proinflammatory and Regulatory Cytokines in Dengue Fever. Indian J. Med. Microbiol..

[29] Jusof F.F., Lim C.K., Aziz F.N., Soe H.J., Raju C.S., Sekaran S.D., Guillemin G.J. (2022). The Cytokines CXCL10 and CCL2 and the Kynurenine Metabolite Anthranilic Acid Accurately Predict Patients at Risk of Developing Dengue With Warning Signs. J. Infect. Dis..

[30] Bethell D.B., Flobbe K., Xuan C., Phuong T., Day N.P.J., Phuong P.T., Buurman W.A., Cardosa M.J., White N.J., Kwiatkowski D. (1998). Pathophysiologic and Prognostic Role of Cytokines in Dengue Hemorrhagic Fever. J. Infect. Dis..

[31] Bozza F.A., Cruz O.G., Zagne S.M., Azeredo E.L., Nogueira R.M., Assis E.F., Bozza P.T., Kubelka C.F. (2008). Multiplex cytokine profile from dengue patients: MIP-1beta and IFN-gamma as predictive factors for severity. BMC Infect. Dis..

[32] Assunção-Miranda I., Amaral F.A., Bozza F.A., Fagundes C.T., Sousa L.P., Souza D.G., Pacheco P., Barbosa-Lima G., Gomes R.N., Bozza P.T. (2010). Contribution of Macrophage Mi-gration Inhibitory Factor to the Pathogenesis of Dengue Virus Infection. FASEB J..

[33] De-Oliveira-Pinto L.M., Marinho C.F., Povoa T.F., De Azeredo E.L., De Souza L.A., Barbosa L.D.R., Motta-Castro A.R.C., Alves A.M.B., Ávila C.A.L., De Souza L.J. (2012). Regulation of Inflammatory Chemokine Receptors on Blood T Cells Associated to the Circulating Versus Liver Chemokines in Dengue Fever. PLoS ONE.

[34] Patro A.R.K., Mohanty S., Prusty B.K., Singh D.K., Gaikwad S., Saswat T., Chattopadhyay S., Das B.K., Tripathy R., Ravindran B. (2019). Cytokine Signature Associated with Disease Severity in Dengue. Viruses.

[35] Puc I., Ho T.-C., Yen K.-L., Vats A., Tsai J.-J., Chen P.-L., Chien Y.-W., Lo Y.-C., Perng G.C. (2021). Cytokine Signature of Dengue Patients at Different Severity of the Disease. Int. J. Mol. Sci..

[36] Bhatt P., Varma M., Sood V., Ambikan A., Jayaram A., Babu N., Gupta S., Mukhopadhyay C., Neogi U. (2024). Temporal cytokine storm dynamics in dengue infection predicts severity. Virus Res..

[37] Bhamarapravati N. (1993). Pathology of Dengue Haemorrhagic Fever. Monograph on Dengue/Dengue Hemorrhagic Fever.

[38] Zompi S., Harris E. (2012). Animal Models of Dengue Virus Infection. Viruses.

[39] Kayesh M.E.H., Tsukiyama-Kohara K. (2021). Mammalian animal models for dengue virus infection: A recent overview. Arch. Virol..

[40] Coronel-Ruiz C., Gutiérrez-Barbosa H., Medina-Moreno S., Velandia-Romero M.L., Chua J.V., Castellanos J.E., Zapata J.C. (2020). Humanized Mice in Dengue Research: A Comparison with Other Mouse Models. Vaccines.

[41] Gubler D.J. (2002). Epidemic dengue/dengue hemorrhagic fever as a public health, social and economic problem in the 21st century. Trends Microbiol..

[42] Azami N.A.M., Takasaki T., Kurane I., Moi M.L. (2020). Non-Human Primate Models of Dengue Virus Infection: A Comparison of Viremia Levels and Antibody Responses during Primary and Secondary Infection among Old World and New World Monkeys. Pathogens.

[43] Vasilakis N., Cardosa J., Hanley K.A., Holmes E.C., Weaver S.C. (2011). Fever from the forest: Prospects for the continued emergence of sylvatic dengue virus and its impact on public health. Nat. Rev. Microbiol..

[44] Onlamoon N., Noisakran S., Hsiao H.-M., Duncan A., Villinger F., Ansari A.A., Perng G.C. (2010). Dengue virus–induced hemorrhage in a nonhuman primate model. Blood.

[45] Omatsu T., Moi M.L., Hirayama T., Takasaki T., Nakamura S., Tajima S., Ito M., Yoshida T., Saito A., Katakai Y. (2011). Common marmoset (Callithrix jacchus) as a primate model of dengue virus infection: Development of high levels of viraemia and demonstration of protective immunity. J. Gen. Virol..

[46] Omatsu T., Moi M.L., Takasaki T., Nakamura S., Katakai Y., Tajima S., Ito M., Yoshida T., Saito A., Akari H. (2012). Changes in hematological and serum biochemical parameters in common marmosets (*Callithrix jacchus*) after inoculation with dengue virus. J. Med. Primatol..

[47] de Barros V.L., de Souza J.T., Costa Z.G.A., de Araujo M.T.F., Braga R.R. (2009). Diagnóstico de Febre Hemorrágica em Primata Não Humano Causado pelo Vírus Dengue: Relato de Caso. Rev. Soc. Bras. Med. Trop..

[48] Ferreira M.S., de Castro P.H.G., Silva G.A., Casseb S.M.M., Júnior A.G.D., Rodrigues S.G., Azevedo R.D.S.d.S., e Silva M.F.C., Zauli D.A.G., Araújo M.S.S. (2014). Callithrix penicillata: A feasible experimental model for dengue virus infection. Immunol. Lett..

[49] Johnson B.W., Russell B.J., Lanciotti R.S. (2005). Serotype-Specific Detection of Dengue Viruses in a Fourplex Real-Time Reverse Transcriptase PCR Assay. J. Clin. Microbiol..

[50] Igarashi A. (1978). Isolation of a Singh’s Aedes albopictus Cell Clone Sensitive to Dengue and Chikungunya Viruses. J. Gen. Virol..

[51] Gubler D.J., Kuno G., Sather G.E., Velez M., Oliver A. (1984). Mosquito Cell Cultures and Specific Monoclonal Antibodies in Surveillance for Dengue Viruses. Am. J. Trop. Med. Hyg..

[52] Ferreira M.S. (2012). Estudo Experimental sobre a Resposta Imunológica em Infecções Sequenciais pelo Vírus Dengue 3 e pelo Vírus Dengue 2 em Primatas Não Humanos da Espécie Callithrix Penicillata.

[53] Travassos da Rosa A.P.A., Travassos da Rosa E.S., Travassos da Rosa J.F.S., Dégallier N., Vasconcelos P.F.C., Rodrigues S.G., Cruz A.C.R. (1994). Os Arbovírus no Brasil: Generalidades, Métodos e Técnicas de Estudo; Documento Técnico nº 2.

[54] Hall W.C., Crowell T.P., Watts D.M., Barros V.L.R., Kruger H., Pinheiro F., Peters C.J. (1991). Demonstration of Yellow Fever and Dengue Antigens in Formalin-Fixed Paraffin-Embedded Human Liver by Immunohistochemical Analysis. Am. J. Trop. Med. Hyg..

[55] Quaresma J.A.S., Barros V.L.R.S., Fernandes E.R., Pagliari C., Takakura C., Vasconcelos P.F.d.C., de Andrade H.F., Duarte M.I.S. (2005). Reconsideration of histopathology and ultrastructural aspects of the human liver in yellow fever. Acta Trop..

[56] Ishak K., Baptista A., Bianchi L., Callea F., De Groote J., Gudat F., Denk H., Desmet V., Korb G., MacSween R.N. (1995). Histological grading and staging of chronic hepatitis. J. Hepatol..

[57] Anthony P.P., Ishak K.G., Nayak N.C., Poulsen H.E., Scheuer P.J., Sobin L.H. (1977). The Morphology of Acute Hepatitis.

[58] MacSween R.N.M., Burt A.D., Portmann B., Hübscher S., Ferrell L., Schmid M. (2012). MacSween’s Pathology of the Liver.

[59] Burt A.D., Ferrell L.D., Hübscher S.G. (2018). Morphology of the Liver.

[60] Lefkowitch J.H. (2015). Anatomic Pathology of the Liver.

[61] Dancey C., Reidy J. (2006). Statistics Without Mathematics for Psychology: Using SPSS for Windows.

[62] Batista P.M., Rocha T.C.D., Andreotti R., Gomes E.C., Silva M.A.N., Svoboda W.K., Nunes J., Guerreiro S.R., Chiang J.O., Vasconcelos P.F.C. (2015). Serosurvey of Arbovirus in Free-Living Non-Human Primates (*Sapajus* spp.) in Brazil. J. Environ. Anal. Chem..

[63] Bhamarapravati N., Tuchinda P., Boonyapaknavik V. (1967). Pathology of Thailand haemorrhagic fever: A study of 100 autopsy cases. Ann. Trop. Med. Parasitol..

[64] Huerre M.R., Lan N.T., Marianneau P., Hue N.B., Khun H., Hung N.T., Khen N.T., Drouet M.T., Huong V.T., Ha D.Q. (2001). Liver Histopathology and Biological Correlates in Five Cases of Fatal Dengue Fever in Vietnamese Children. Virchows Arch..

[65] Aye K.S., Charngkaew K., Win N., Wai K.Z., Moe K., Punyadee N., Thiemmeca S., Suttitheptumrong A., Sukpanichnant S., Prida M. (2014). Pathologic highlights of dengue hemorrhagic fever in 13 autopsy cases from Myanmar. Hum. Pathol..

[66] Moragas L.J., Alves F.d.A.V., Oliveira L.d.L.S., Salomão N.G., Azevedo C.G., da Silva J.F.R., Basílio-De-Oliveira C.A., Basílio-De-Oliveira R., Mohana-Borges R., de Carvalho J.J. (2023). Liver immunopathogenesis in fatal cases of dengue in children: Detection of viral antigen, cytokine profile and inflammatory mediators. Front. Immunol..

[67] Ribeiro Y.P., Falcão L.F.M., Smith V.C., de Sousa J.R., Pagliari C., Franco E.C.S., Cruz A.C.R., Chiang J.O., Martins L.C., Nunes J.A.L. (2023). Comparative Analysis of Human Hepatic Lesions in Dengue, Yellow Fever, and Chikungunya: Revisiting Histopathological Changes in the Light of Modern Knowledge of Cell Pathology. Pathogens.

[68] Costa D.S., Moita L.A., Alves E.H.P., Sales A.C.S., Rodrigues R.R.L., Galeno J.G., Gomes T.N., Ferreira G.P., Vasconcelos D.F.P. (2019). Dengue Virus and Yellow Fever Virus Damage the Liver: A Systematic Review About the Histopathological Profiles. J. Gastroenterol. Hepatol. Res..

[69] Couvelard A., Marianneau P., Bedel C., Drouet M.T., Vachon F., Hénin D., Deubel V. (1999). Report of a fatal case of dengue infection with hepatitis: Demonstration of dengue antigens in hepatocytes and liver apoptosis. Hum. Pathol..

[70] Torres E.M. (2005). Dengue.

[71] Bradham C.A., Qian T., Streetz K., Trautwein C., Brenner D.A., Lemasters J.J. (1998). The Mitochondrial Permeability Transition Is Required for Tumor Necrosis Factor Alpha-Mediated Apoptosis and Cytochrome *c* Release. Mol. Cell. Biol..

[72] Attisano L., Wrana J.L. (2002). Signal Transduction by the TGF-β Superfamily. Science.

[73] Wrana J.L. (2002). Phosphoserine-Dependent Regulation of Protein-Protein Interactions in the Smad Pathway. Structure.

[74] Quaresma J.A., Barros V.L., Pagliari C., Fernandes E.R., Guedes F., Takakura C.F., Andrade H.F., Vasconcelos P.F., Duarte M.I. (2006). Revisiting the liver in human yellow fever: Virus-induced apoptosis in hepatocytes associated with TGF-β, TNF-α and NK cells activity. Virology.

[75] Perez A.B., Sierra B., Garcia G., Aguirre E., Babel N., Alvarez M., Sanchez L., Valdes L., Volk H.D., Guzman M.G. (2010). Tumor necrosis factor–alpha, transforming growth factor–β1, and interleukin-10 gene polymorphisms: Implication in protection or susceptibility to dengue hemorrhagic fever. Hum. Immunol..

[76] Abbas A.K., Lichtman A.H., Pillai S. (2023). Imunologia Celular e Molecular.

[77] Santos N.S.O., Romanos M.T.V., Wigg M.D. (2008). Introdução à Virologia Humana.

[78] Castera L., Pawlotsky J.-M. (2005). Noninvasive diagnosis of liver fibrosis in patients with chronic hepatitis C. Medscape Gen. Med..

[79] Dev A., Patel K., McHutchison J.G. (2004). Hepatitis C and steatosis. Clin. Liver Dis..

[80] Alberts B., Johnson A., Lewis J., Raff M., Roberts K., Walter P. (2002). Molecular Biology of the Cell.

[81] Barth O.M., Barreto D.F., Paes M.V., Takiya C.M., Pinhão A.T., Schatzmayr H.G. (2006). Morphological studies in a model for dengue-2 virus infection in mice. Mem. Inst. Oswaldo Cruz.

[82] Nguyen T., Nguyen T., Tieu N. (1997). The impact of dengue haemorrhagic fever on liver function. Res. Virol..

[83] de Souza L.J., Nogueira R.M.R., Soares L.C., Soares C.E.C., Ribas B.F., Alves F.P., Vieira F.R., Pessanha F.E.B. (2007). The impact of dengue on liver function as evaluated by aminotransferase levels. Braz. J. Infect. Dis..

[84] Nascimento D.D., de Castro A.R.C.M., Froes Í.B., Bigaton G., de Oliveira É.C.L., Fabbro M.F.J.D., da Cunha R.V., da Costa I.P. (2011). Clinical and laboratory findings in patients with dengue associated with hepatopathy. Rev. Soc. Bras. Med. Trop..

[85] Kuo C.-H., Tai D.-I., Chang-Chien C.-S., Lan C.-K., Chiou S.-S., Liaw Y.-F. (1992). Liver Biochemical Tests and Dengue Fever. Am. J. Trop. Med. Hyg..

[86] Lee L.K., Gan V.C., Lee V.J., Tan A.S., Leo Y.S., Lye D.C. (2012). Clinical relevance and discriminatory value of elevated liver ami-notransferase levels for dengue severity. PLoS Negl. Trop. Dis..

[87] Wang X.-J., Wei H.-X., Jiang S.-C., He C., Xu X.-J., Peng H.-J. (2016). Evaluation of aminotransferase abnormality in dengue patients: A meta analysis. Acta Trop..

[88] Brasil R.A. (2005). Caracterização do Fenótipo da Resposta Inflamatória, da Expressão de Citocinas e do Mecanismo de Morte Celular no Fígado de Pacientes com Febre Hemorrágica da Dengue.

[89] Jungermann K., Katz N. (1989). Functional specialization of different hepatocyte populations. Physiol. Rev..

[90] Park D.R., Thomsen A.R., Frevert C.W., Pham U., Skerrett S.J., Kiener P.A., Liles W.C., Park D.R. (2003). Fas (CD95) Induces Proinflammatory Cytokine Responses by Human Monocytes and Monocyte-Derived Macrophages. J. Immunol..

[91] Cummings R.J., Barbet G., Bongers G., Hartmann B.M., Gettler K., Muniz L., Furtado G.C., Cho J., Lira S.A., Blander J.M. (2016). Different tissue phagocytes sample apoptotic cells to direct distinct homeostasis programs. Nature.

[92] Quaresma J.A., Barros V.L., Pagliari C., Fernandes E.R., Andrade H.F., Vasconcelos P.F., Duarte M.I. (2007). Hepatocyte lesions and cellular immune response in yellow fever infection. Trans. R. Soc. Trop. Med. Hyg..

[93] Quaresma J.A.S., Pagliari C., Medeiros D.B.A., Duarte M.I.S., Vasconcelos P.F.C. (2013). Immunity and immune response, pathology and pathologic changes: Progress and challenges in the immunopathology of yellow fever. Rev. Med Virol..

[94] Kurane I., Kontny U., Janus J., Ennis F.A. (1990). Dengue-2 virus infection of human mononuclear cell lines and establishment of persistent infections. Arch. Virol..

[95] Liu Z.-X., Govindarajan S., Okamoto S., Dennert G. (2000). NK Cells Cause Liver Injury and Facilitate the Induction of T Cell-Mediated Immunity to a Viral Liver Infection. J. Immunol..

[96] Scroferneker M.L., Pohlmann P.R. (1998). Imunologia: Básica e Aplicada.

[97] Huang Y.-H., Liu C.-C., Wang S.-T., Lei H.-Y., Liu H.-S., Lin Y.-S., Wu H.-L., Yeh T.-M. (2001). Activation of coagulation and fibrinolysis during dengue virus infection. J. Med. Virol..

[98] Liao B., Tang Y., Hu F., Zhou W., Yao X., Hong W., Wang J., Zhang X., Tang X., Zhang F. (2015). Serum levels of soluble vascular cell adhesion molecules may correlate with the severity of dengue virus-1 infection in adults. Emerg. Microbes Infect..

[99] Blissard G.W., Wenz J.R. (1992). Baculovirus gp64 Envelope Glycoprotein Is Sufficient To Mediate pH-Dependent Membrane Fusion. J. Virol..

[100] Inyoo S., Suttitheptumrong A., Pattanakitsakul S.-N. (2017). Synergistic Effect of TNF-α and Dengue Virus Infection on Adhesion Molecule Reorganization in Human Endothelial Cells. Jpn. J. Infect. Dis..

[101] Yang K.D., Yeh W.T., Yang M.Y., Chen R.F., Shaio M.F. (2001). Antibody-dependent enhancement of heterotypic dengue infections involved in suppression of IFN-γ production. J. Med. Virol..

