# Supplementary material for: Sequential Dengue Virus Infection in Marmosets: Histopathological and Immune Responses in the Liver
Source: Viruses. 2025 Dec 15;17(12):1619. doi: 10.3390/v17121619 (PMC12737778; doi:10.3390/v17121619)
Supplement: Supplementary file 1 [file viruses-17-01619-s001.zip › viruses-4000130-supplementary.pdf]

Supplementary Materials

Table S1 – Scores parameters and characterization applied to the morphological analysis of the liver.

Parameters*	Score description
Hepatocytes apoptosis (number of cells)	0: ≤ 3; 1: 4 to 14; 2: 15 to 28; 3: ≥ 28. Analysis in ≥ 50 fields/40x.
Hepatocelular necrosis (number of cells)	0: ≤ 3; 1: 4 to 14; 2: 15 to 28; 3: ≥ 28. Analysis in ≥ 50 fields/40x.
Lobular necrosis	0: absence; 1: injury extends 1/3 of HCV to portal space; 2: lesion extends 2/3 of HCV to portal space; 3: lesion extends throughout the acinus. Analysis in ≥ 50 fields/40x
Swelling	0: absence; 1: injury extends 1/3 of HCV to portal space; 2: lesion extends 2/3 of HCV to portal space; 3: lesion extends throughout the acinus. Analysis in ≥ 50 fields /40x
Steatosis	0: absence; 1: injury extends 1/3 of HCV to portal space; 2: lesion extends 2/3 of HCV to portal space; 3: lesion extends throughout the acinus. Analysis in ≥ 50 fields /40x
Küpffer cells hyperplasia (cells / field)	0: absence; 1: 3 to 5; 2: 6 to 10; 3:> 10. Analysis in ≥ 50 fields /40x
Hemosiderin	0: absence; 1: 3 to 5 cells / field; 2: 6 to 10 cells / field; 3:> 10 cells / field. Analysis in ≥ 50 fields /40x
Lobular inflammatory infiltrate (foci of inflammatory cells)	0: up to 3; 1: 4 up to 15; 2: 16 up to 50; 3: above 50. Analysis in ≥ 50 fields /40x
Portal space (PS) inflammatory infiltrate (number of inflammatory cells)	0: rare; 1: few; 2: moderate; 3: intense. Analysis in 10 random PS.
Central hepatic vein (CHP) inflammatory infiltrate (number of inflammatory cells)	0: absence; 1: few; 2: moderate; 3: intense. Analysis in 10 random CHP
Reticulinic hash injury (foci of necrosis)	0: no collapse; 1: few; 2: moderate; 3: lesion extends throughout the acinus. Analysis in ≥ 50 fields / 40x.
Hemorrhage	0: absence; 1: discrete; 2: moderate; 3: intense. Analysis in ≥ 50 fields / 40x.
Congestion	0: absence; 1: few; 2: moderate; 3: intense. Analysis in ≥ 50 fields / 40x.
Ductular proliferation	0: absence; 1: few; 2: moderate; 3: intense. Analysis in ≥ 50 fields / 40x.
Fibrosis	0: absence; 1: few; 2: moderate; 3: intense. Analysis in ≥ 50 fields / 40x.

Table S2. Antibodies used for the characterization of the immunophenotype and cytokines expression on the liver of Callithrix penicillata after sequential DENV infection.

**Antibody (catalog number)**	**Manufacturer**	**Working dilution**	**Investigation**
Anti-Lysozyme (A0099)	Dako	1:2.000	Inflammatory response
Anti – S100 (Z0311)	Dako	1:800	Inflammatory response
Anti– CD20 (M755)	Dako	1:300	Inflammatory response
Anti -CD4 (NCL-L-CD4-1F6)	Novocastra	1:40	Inflammatory response
Anti – CD57 (MS-136-P)	Neomarkes	1:50	Inflammatory response
Anti-CD95 (M 3555)	Dako	1:30	Apoptosis
Anti –TNF alpha (AF210NA)	R&D systems	1:20	Cytokine expression
Anti – IFN gamma (MAB285)	R&D systems	1:30	Cytokine expression
Anti-TGF beta (SC 82)	Santa Cruz	1:400	Cytokine expression
Anti – IL8 (AF 208NA)	R&D systems	1:25	Cytokine expression
Anti – IL10 (MAB217)	R&D systems	1:10	Cytokine expression
Anti-VCAM1 (BBA19)	R&D systems	1:300	Cytokine expression

Table S3. Immunohistochemical evaluation - mean and standard deviation of the number of positive cells/mm2 in the hepatic acinus by groups.

Antibody	NC PI	Acute PI	Convalescent PI	NC SI	Acute SI	Convalescent SI
VCAM	114.25 ±37.87	133.36 ±199.92	163.64 ±234.99	15.11 ±10.09	103.23 ±146.17	97.58 ±116.90
S100	28.45 ±16.29	60.61 ±88.84	58.85 ±19.92	38.52 ±16.80	56.26 ±36.12	76.35 ±36.15
IL-10	9.78 ±3.08	46.08 ±36.81	21.06 ±16.44	9.33 ±8.37	22.26 ±12.30	17.33 ±9.82
TGF-β	9.48 ±5.43	93.63 ±94.12	13.83 ±10.34	30.81 ±3.70	28.56 ±28.53	52.39±27.24
CD95	0.00 ±0.00	336.34 ±80.17	2.54±2.90	0.00 ±0.00	1.54±2.84	0.24±0.63
CD4	0.00 ±0.00	2.54 ±2.95	0.85 ±1.33	0.00 ±0.00	0.83 ±2.31	0.12±0.46
TNF-α	13.04 ±2.72	135.18 ±114.24	137.33 ±75.19	116.98 ±25.65	164.03 ±194.11	205.58 ±120.36
IFN-ɤ	5.93 ±6.24	41.20 ±17.39	47.58 ±43.27	33.93 ±12.06	38.68 ±25.40	48.31 ±28.74
NK	0.59 ±1.03	9.80 ±10.66	4.20±3.88	0.89 ±1.54	4.78 ±5.45	1.69±2.10
IL-8	3.85 ±4.01	3.14 ±4.82	3.96±5.40	0.00 ±0.00	1.07 ±1.43	0.89±1.54
CD20	9.93 ±2.53	43.26 ±25.99	22.60 ±10.62	20.44 ±3.20	39.22 ±30.86	27.93 ±10.40
Lysozyme	180.80 ±99.45	336.34 ±80.17	426.13 ±108.08	337.28 ±90.85	283.22 ±144.75	514.92 ±113.55

Notes: NC PI = control animal of primary infection; Acute PI = acute phase of primary infection; Convalescent PI = convalescent phase of primary infection; NC SI= control animal of secondary infection; Acute SI = acute phase of secondary infection; Convalescent SI = convalescent phase of secondary infection.

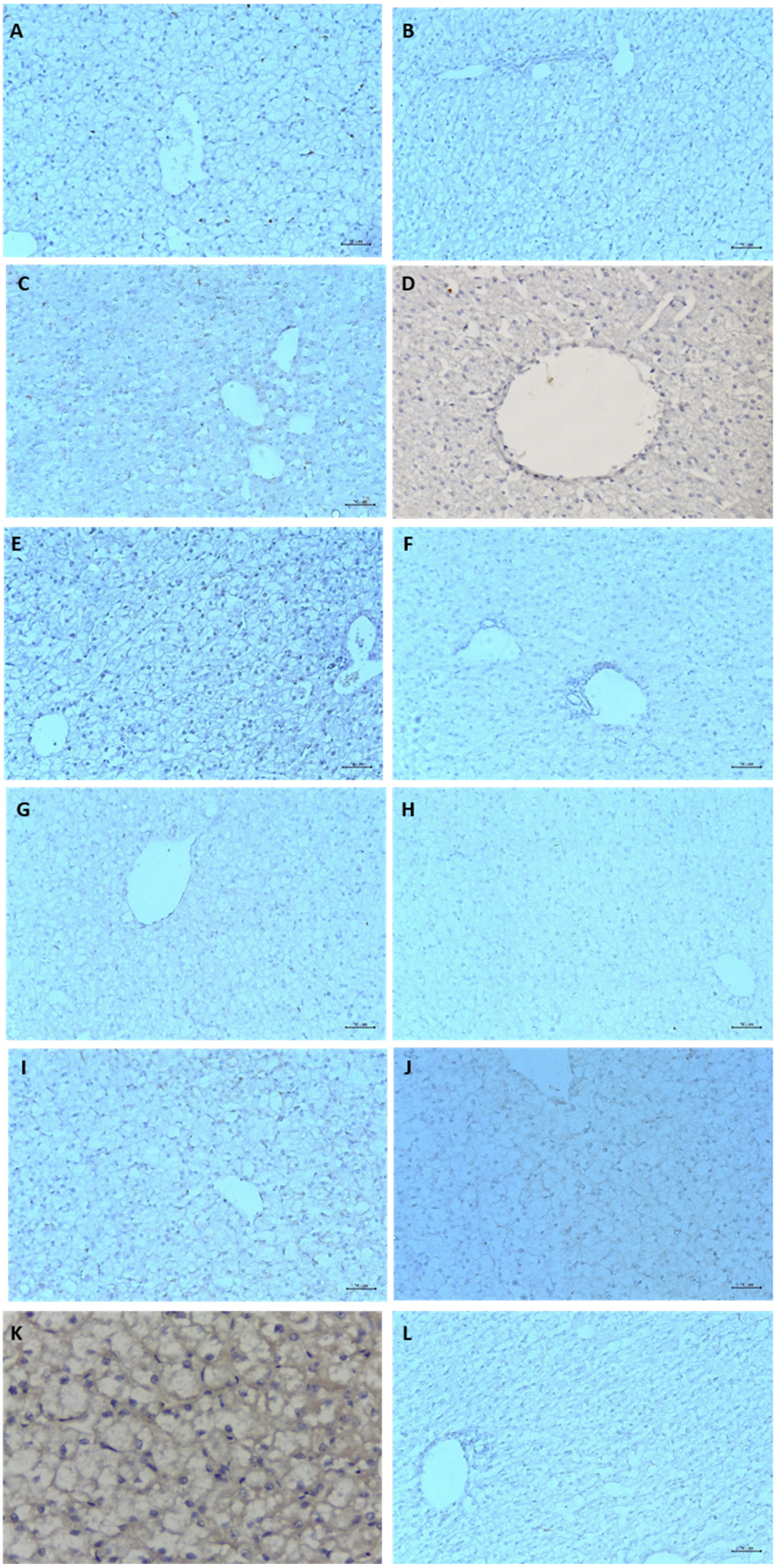

Figure S1. Immunohistochemistry for the various markers in the hepatic parenchyma of the negative control (Callithrix penicillata) for (A) Lysozyme; (B) NK; (C) S100; (D) CD20; (E) CD4; (F) CD95; (G) TNF-α; (H) IL-8; (I) TGF-β; (J) IL-10; (K) IFN-γ; (L) VCAM.
